# Proteomics Analysis Reveals the Molecular Mechanism of MoPer1 Regulating the Development and Pathogenicity of *Magnaporthe oryzae*

**DOI:** 10.3389/fcimb.2022.926771

**Published:** 2022-06-24

**Authors:** Yue Chen, Xiyang Wu, Chunyan Chen, Qiang Huang, Chenggang Li, Xin Zhang, Xinqiu Tan, Deyong Zhang, Yong Liu

**Affiliations:** ^1^ State Key Laboratory of Hybrid Rice and Institute of Plant Protection, Hunan Academy of Agricultural Sciences, Changsha, China; ^2^ Long Ping Branch, Graduate School of Hunan University, Changsha, China; ^3^ College of Plant Protection, Hunan Agricultural University, Changsha, China

**Keywords:** MoPer1, proteomics, differentially expressed proteins, pathogenic protein, qRT-PCR validation

## Abstract

Glycosylphosphatidylinositol (GPI) anchoring the protein GPI modification post-transcriptionally is commonly seen. In our previous study, MoPer1, a GPI anchoring essential factor, has a critical effect on *Magnaporthe oryzae* growth, pathogenicity, and conidiogenesis, but its molecular mechanism is not clear. Here, we extracted the glycoproteins from the Δ*Moper1* mutant and wild-type Guy11 to analyze their differential levels by quantitative proteomic analysis of TMT markers. After background subtraction, a total of 431 proteins, with significant changes in expression, were successfully identified, and these differential proteins were involved in biological regulation, as well as cellular process and metabolic process, binding, catalytic activity, and other aspects. Moreover, we found that MoPer1 regulates the expression of 14 proteins involved in growth, development, and pathogenicity of *M. oryzae*. The above findings shed light on MoPer1’s underlying mechanism in regulating growth, development, and pathogenicity of *M. oryzae*.

## Introduction

Cell wall is an organelle unique to fungi, and has a critical effect on keeping an inherent. At the same time, it plays an important role in maintaining normal growth processes such as physiological cellular metabolism, osmotic pressure, and ion exchange (Ahrazem et al., 2007). Protein and polysaccharides such as chitin and glucan are major fungal cellular components. Studies have found that after transcription and translation, some cell wall proteins must be anchored by glycosylphosphatidylinositol (GPI) before they can bind to the cell wall and perform their normal biological functions (Bowman and Free, 2010). In *Saccharomyces cerevisiae*, more than 60 genes encoding GPI anchor proteins have been found (Kapteyn et al., 1999). According to their location, GPI anchor proteins are divided into two categories. One type is GPI-mannose protein bound to the polysaccharide on the cell wall, which is mainly involved in the following biological processes: fungus adhesion, infective hypha growth, filamentation, coagulation, and mating (Guo et al., 2000). The other type is composed of proteins with enzymatic activity, such as glucanase; these proteins are necessary for cell wall biosynthesis (Gastebois et al., 2010). The research results showed that proteins anchored by GPI have essential effects on the growth, development, and integrity of yeast cell wall. Therefore, the study of these proteins has also clarified the key role of GPI anchored in the growth and development of fungi.

The GPI anchor structure generally has 5 core components: alcoholamine phosphate, mannose, glucosamine, and phosphatidylinositol. The GPI anchoring structure is generated within the endoplasmic reticulum (ER), and its lipid groups experience reconstruction in ER by diverse modification processes, when it combines with its target protein. It mostly consists of 3 processes: first, deacylation of inositol, then formation of lyso-GPI through cutting off acyl chain on diacylglycerol’s sn-2 position, and finally connection of the saturated 26-carbon acyl group at the sn-2 position (Orlean and Menon, 2007; Fujita and Jigami, 2008; Fujita and Kinoshita, 2012). In mammals, together with the model fungus *S. cerevisiae*, the genes related to GPI anchoring were well studied. In *S. cerevisiae*, these 3 steps are respectively completed under the action of inositol deacylase Bst1, GPI phospholipase Per1, and Gup1 protein. Studies have found that the lack of *BST1* delays the formation of GPI-anchored proteins (Fujita et al., 2006), while deletion of *PER1* gene inhibits the transport of yeast GPI-anchored protein (Fujita et al., 2007); at the same time, the deletion of two genes can affect the integrity of yeast cell wall. The results of these series of studies indicate that the yeast GPI anchor structure has a critical effect on the GPI-anchored protein maturation, transport, as well as cell wall adhesion. Although the types and biological functions of GPI-anchored related proteins have been preliminary research results in yeast, relevant research in plant pathogenic fungi has not been reported.

In our previous study, the functions of MoPer1, a homolog of yeast ScPer1, was characterized in *Magnaporthe oryzae*. We found that the *MoPER1* gene was significantly upregulated in the conidia and infection stages of *M. oryzae*, and the Δ*Moper1* mutant was defective in vegetative growth, conidiation, appressoria formation, cell wall integrity, and host infection (Chen et al., 2018).

To shed further light on the role of *MoPER1*, we used quantitative proteomics to analyze changes in protein expression in the Δ*Moper1* mutants and elucidate the molecular mechanism of MoPer1 regulating the development and pathogenicity of *M. oryzae*.

## Materials and Methods

### Glycoprotein Extraction of *M. oryzae*

*M. oryzae* Guy11 and Δ*Moper1* mutant strains were used in this study. Referring to previous studies to obtain fungal mycelium in liquid CM for total protein extraction, (Liu et al., 2020), glycoprotein was extracted by the FOCUS™ Glycoprotein kit (G-Biosciences, USA) according to the manufacturer’s instructions. The BCA (bicinchoninic acid) method was utilized to determine glycoprotein concentration, followed by preservation under −80°C until subsequent use.

### Separation by SDS-PAGE

After being collected in every sample, proteins (20 mg) were mixed with 5× loading buffer that contained 0.5% bromophenol blue, 10% SDS, 50% glycerol, 250 mM Tris-HCl, and 500 mM DTT. Thereafter, the mixture was boiled within the water bath for a 5-min period before 90 min of 12.5% SDS-PAGE (at 14 mA). Lastly, Coomassie blue was used to stain the gel (Hiroyuki et al., 2019).

### Trypsin Digestion

In total, 50 μg of glycoprotein from each sample was diluted with lysis buffer to adjust to the same concentration and volume. DTT was added to the above glycoprotein solution to attain the eventual content of DTT at 5 mM, and mixed, followed by 30 min of incubation under 55°C and later cooling on ice to ambient temperature. The corresponding iodoacetamide (IAA) quantity was added to make 10 mM of the final concentration, and mixed sufficiently before 15-min preservation under ambient temperature in the dark. Acetone (6-fold volume) was added to precipitate glycoprotein prior to overnight preservation under −20°C and then centrifuged at 4°C to collect the precipitate. One hundred microliters of 200 mM tetraethylammonium (TEAB) was added to reconstitute the pellet. Then, Trypsin-TPCK (1 mg/ml) was added at 1/50 sample weight for overnight digestion under 37°C.

### Tandem Mass Tag Labeling

This work carried out six-plex TMT labeling (Thermo Fisher Scientific Inc., San Jose, CA) in line with specific protocols. After lyophilization, 100 mM TEAB buffer (50 μl) was used to resuspend samples and vortexed to mix, and the labeling reaction was performed (Bradley et al., 2019). The TMT reagent was removed from the refrigerator and equilibrated to room temperature; 88 μl of anhydrous acetonitrile was added, followed by 5-min vortexing and centrifugation. The TMT reagent (41 μl) was added into the sample, vortexed to mix, and stood for a 1-h period under ambient temperature; 5% hydroxylamine (8 μl) was added for a 15-min period to stop the reaction. The CK group (Guy11) was labeled with 126, 127, and 128. The T group (Δ*Moper1* mutant) was labeled with 129, 130, and 131.

### RPLC Analysis

This work adopted the 1100 HPLC System (Agilent) to separate RP with the use of the 5-μm Agilent Zorbax Extend RP column (150 mm × 2.1 mm). Later, RP gradient was conducted using mobile phases A (which contained 2% acetonitrile within the HPLC water) and B (which contained 98% acetonitrile within the HPLC water). Gradient elution conditions were as follows: 8% A in 0–8 min, 98%–95% A in 8–8.01 min, 95%–75% A in 8.01–48 min, 75%–60% A in 48–60 min, 60%–10% A in 60–60.01 min, 10% A in 60.01–70 min, 10%–98% A in 70–70.01 min, and 98% A in 70.01–75 min. After separation at the 300 μl/min fluent flow rate, we observed tryptic peptides at 210 and 280 nm. At 8–60 min later, each sample was harvested, followed by the collection of respective eluents at 1-min intervals from centrifugal tubes 1–15. Sample recycling was conducted in the above order until the completion of gradient. Mass spectrometry (MS) was later conducted on those isolated peptides.

### Mass Spectrometry

This work adopted the Q-Exactive HF mass spectrometer (Thermo, USA) for MS analysis through using the Nanospray Flex source (Thermo, USA). Furthermore, the C18 column (15 cm × 75 μm) was utilized to load and separate samples using the EASY-nLCTM 1200 system (Thermo, USA). We set the linear gradient and flow rate at 75 min (5%–45% B in 0–63 min, 45%–90% B in 63–65 min, 90% B in 65–67 min; mobile phase A consisted of 0.1% FA contained within water, whereas mobile phase B included 0.1% FA supplemented within ACN) and 300 nl/min, separately.

This work later obtained full MS scans within the 350–1500 m/z mass range with the AGC target value and mass resolution being 3e6 and 60,000, respectively. From MS spectra, we fragmented 20 peaks with the strongest intensity through higher-energy collisional dissociation (HCD), and the collision energy was 30. The following parameters were used to obtain MS/MS spectra: resolution = 15,000, max injection time = 40 ms, and AGC target = 2e5. Using the positive mode, this work set Q Exactive HF dynamic exclusion at 30.0 s.

### Identification and Measurement of Proteins

We utilized Proteome Discoverer (v.2.4) for searching relevant raw data thoroughly from *M. oryzae* genome database (https://fungidb.org/fungidb/app/record/organism/NCBITAXON_242507) using Trypsin digestion specificity. During this process, this work regarded alkylation on cysteine to be the fixed modifications. For protein quantitation, 2 or more specific spectra were obtained upon the threshold of false discovery rate (FDR) < 0.01. In the meantime, this study determined differentially expressed proteins (DEPs) upon the thresholds of *p* < 0.05 and fold change (FC) ≤0.83 or ≥1.2.

### Gene Ontology Function Notes as Well as Kyoto Encyclopedia of Genes and Genomes Pathway Notes

After obtaining DEPs, GO/KEGG enrichment analysis was performed on the differential proteins, and their functions were described. GO/KEGG function enrichment analysis method is as follows: use the species protein as the background list, then use the difference protein list as the candidate list filtered based on the background list, and determine the significant enrichment of the representative function set in the difference protein list using the hypergeometric distribution test. Later, *p*-value is adjusted by the Benjamini–Hochberg multiple test for obtaining FDR. The GO of adjusted *p* < 0.05 indicates significance. KEGG is a main public database for systematic analysis of protein metabolic pathways in cells (Bowman and Free, 2010). Use the KEGG database to analyze the differential proteins (combined with the KEGG annotation results), and determine whether DEPs are significantly enriched in all pathway entries by the hypergeometric distribution test, expressed as *p*-value. Data were later selected upon threshold of adjusted *p* < 0.05.

### Primer Design and qRT-PCR Analysis

This work utilized TRIzol Reagent (Invitrogen, Carlsbad, CA, USA) for isolating total RNA in every sample, while total RNA content and purity were detected by the nano-drop spectrophotometer (Thermo Fisher Scientific). Later, this work chose six key genes of *M. oryzae* to detect gene levels through qRT-PCR. Later, nucleotide sequences were obtained from the *M. oryzae* genome database. In every candidate, we adopted Primer Express v3.0 software (Applied Biosystems) for designing gene primers.

For RT-PCR, this work prepared first-strand cDNA from 5 μg of total RNA with the Reverse Transcription Kit (Transgen biotech) through reverse transcription. By adopting the ABI PRISM 7700 Sequence Detection System (Applied Biosystems), this work carried out qRT-PCR on 3 individual samples with SYBR Green PCR Master Mix in a 20-μl PCR system that contained cDNA template as well as gene-specific primers. The gene MGG_03982 with stable ACTIN expression was subject to amplification using FL4738 and FL4739 primers (**Supplementary Table 3**) for normalizing and relative quantification (2^−Δ;Δ;Ct^).

### Bioinformatics Analysis

This work utilized OmicsBean software (http://www.omicsbean.com:88/) for GO, KEGG, and hierarchical cluster (HCL) analyses. N-Glycosylation site prediction was carried out *via* the URL: https://services.healthtech.dtu.dk/service.php?NetNGlyc-1.0

## Results

### SDS-PAGA

**Figure 1:**
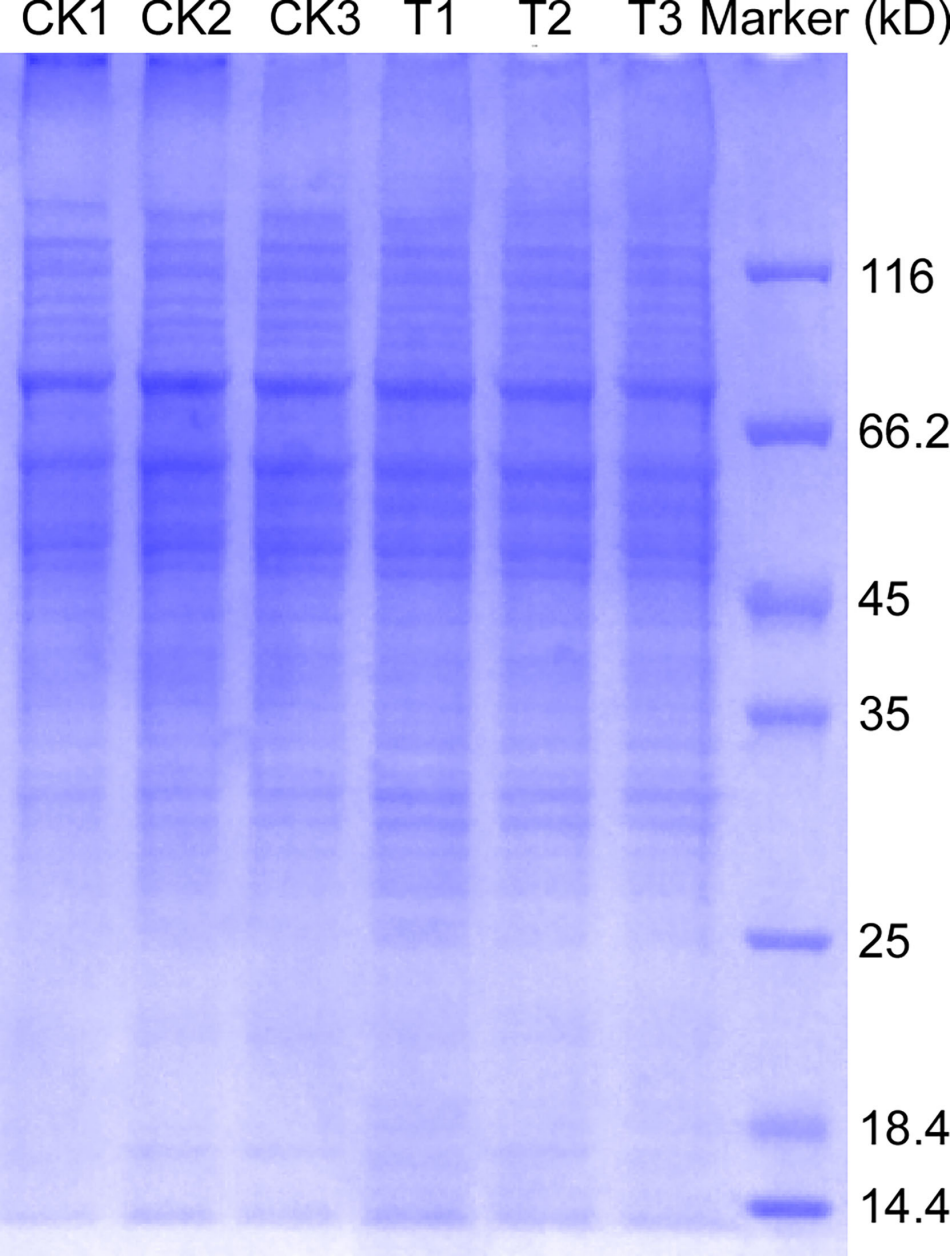
Glycoprotein extraction and SDS-PAGE. The total protein of *M. oryzae* was extracted and purified by the FOCUS™ Glycoprotein kit, and then sodium dodecyl sulfate polyacrylamide gel electrophoresis was performed. T: the Δ*Moper1* mutant.

We carried out a preliminary experiment to ensure that the TMT proteomics quantitative test was smoothly conducted. SDS-PAGE was performed to analyze glycoprotein extracts from *M. oryzae* Guy11 and Δ*Moper1* mutant strains. The results showed that 6 samples had bright protein bands and high total protein levels (**Figure 1**); after the normal operation of the preliminary experiment, we carried out follow-up experiments.

### Protein Identification and Quantification

**Figure 2:**
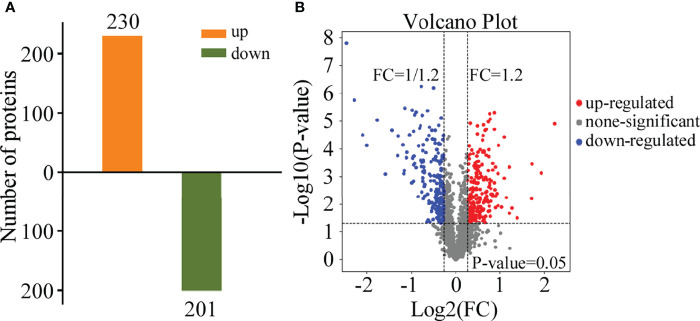
Differential expression protein (DEP) analysis. **(A)** Quantitative identifications of upregulated and downregulated proteins by TMT. **(B)** Distribution of the total identified protein is shown by the volcano plot. The *x*-axis shows various fold change groups and the *y-*axis shows the *p-*value.

**Figure 3:**
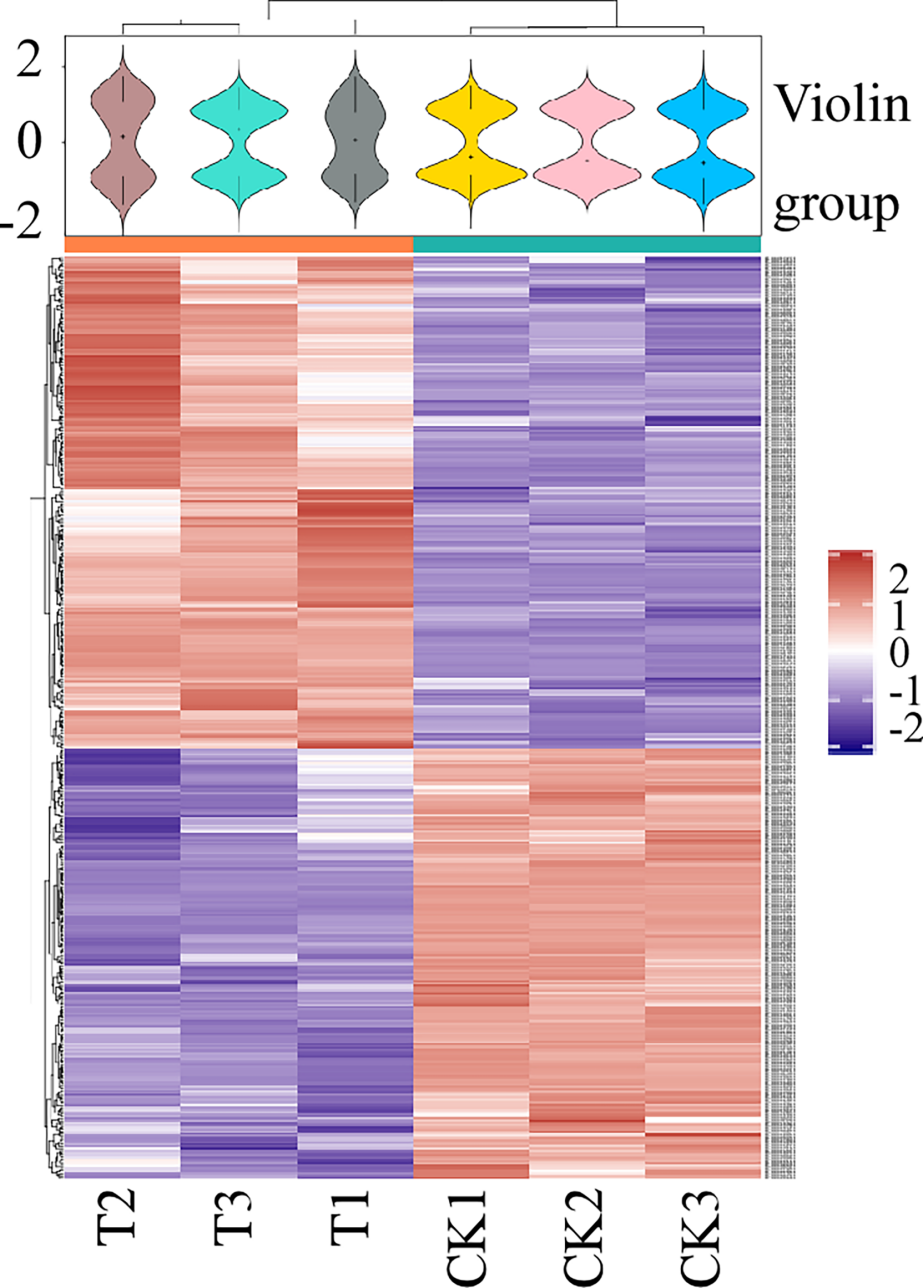
Hierarchical clustering was used to analyze the DEPs. Heat map shows the expression patterns of different groups. Red means a high relative expression level, whereas blue means a low relative expression level.

To obtain data overview, we carried out LC-MS/MS and TMT labeling on glycoproteins to compare their levels between *M. oryzae*, the Δ*Moper1* mutant, and Guy11 strain. Proteome Discover 2.4 was applied in searching and analyzing the acquired data. We obtained altogether 2,766 proteins by 1 or more specific peptides of ≥95% confidence (**Supplementary Table 1**). Moreover, there were altogether 431 DEPs between the Δ*Moper1* mutant and Guy11 strain, as shown in **Supplementary Table 2**. Of these DEPs, 230 showed upregulation, whereas 201 showed downregulation (**Figure 2A**). Moreover, according to the volcano plot, differentially abundant proteins (DAPs) were mostly distributed in 1.2–2.0 fold change (FC), and only a few of them showed >2.0 FC (**Figure 2B**). As revealed by hierarchical clustering analysis, when judging based on *p*-value < 0.05 and FC <0.83 or >1.2, the protein expression of the differentiated group exhibited significant difference compared with the undifferentiated group (**Figure 3**).

### Functional Classification for DEPs

**Figure 4:**
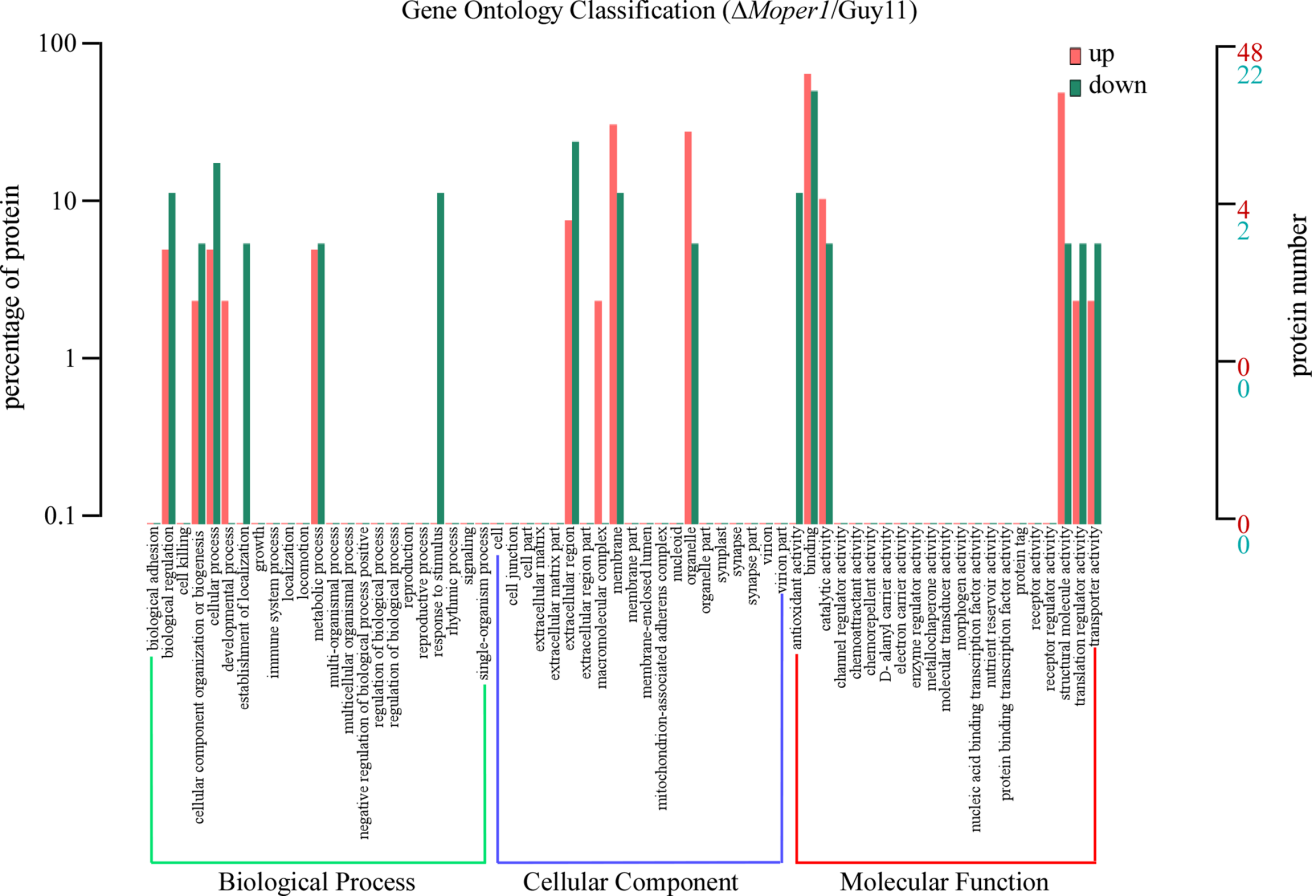
Comparison of the level of distribution of DEPs in GO terms. Red indicates upregulated GO entries enriched by DEPs, green indicates downregulated GO entries enriched by DEPs, the horizontal axis denotes the fixed number of entries, and the vertical axis indicates the protein number and percentage of the corresponding entry.

The GO entries corresponding to DEPs quantity >1 were screened from 3 categories, and then 10 entries were sorted in ascending order according to the −log10 *p*-value corresponding to each entry (**Figure 4**). In brief, based on classification analysis, DEPs mostly participated in biological process (BP), cellular component (CC), and molecular function (MF). In BP, the DEPs were implicated in cellular component organization or biogenesis, biological regulation, metabolic process, and cellular process. With regard to the CC category, DEPs were enriched in extracellular region, membrane, organelle, etc. In MF, DEPs were mainly enriched in catalytic activity, binding, translation regulator activity, and structural molecule activity.

**Figure 5:**
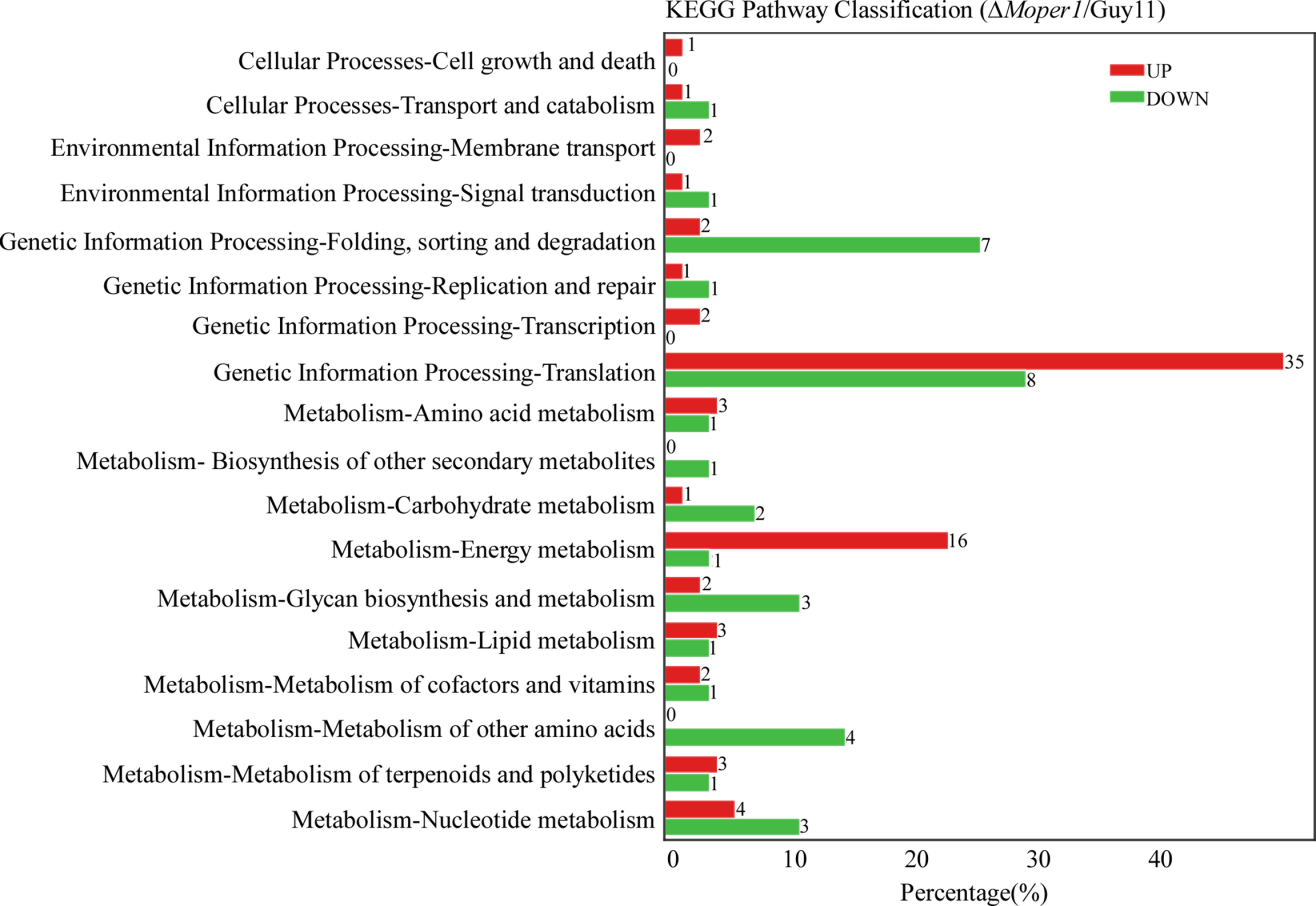
Comparison of the level of distribution of DEPs in the KEGG pathway. The horizontal axis denotes the ratio of the DEPs annotated to the KEGG pathway, the vertical axis represents the name of the pathway, and the numbers on the right side of the column represent the number of DEPs annotated to this pathway.

By analyzing the level of distribution of DEPs in the KEGG pathway, there were 18 entries, mostly related to processing of environmental information, cell process, metabolism, and processing of genetic information (**Figure 5**). The entry with the most DEPs was translation, including 35 with upregulation and 8 with downregulation (**Figure 5**). Additionally, we found 5 DEPs belonging to glycan biosynthesis and metabolism and 3 DEPs belonging to carbohydrate metabolism (**Figure 5**), which indicates that MoPer1 may be involved in the synthesis and metabolism of carbohydrates of *M. oryzae*.

### Moper1 Regulates the Expression of Proteins Involved in Pathogenicity

Considering that the Δ;*Moper1* mutant reduced pathogenicity, we searched the proteome database for the reported proteins involved in growth, development, and pathogenicity regulated by MoPer1. Among MoPer1 regulated genes, we found that there were 14 proteins involved in growth and pathogenicity in *M. oryzae*, including 7 downregulated proteins and 7 upregulated proteins (**Table 1**). We further predicted whether these proteins have N-glycosylation sites. Interestingly, except for MoRsy1 and MoMnh6, the remaining 12 proteins all contained potential N-glycosylation sites (**Table 1**).

**Table 1:** TMT data for proteins known as pathogenicity factors.

Gene_ID	Description	Fold change(Δ*Moper1*/WT)	p-Value	Reference	Predicted N-glycosylation sites ^(a)^
MGG_00527	Extracellular Matrix Protein MoEmp1	0.48	3.06E-05	Ahn et al., 2004	Yes
MGG_09912	Calcium/calmodulin-dependent kinase MoCmk1	0.60	0.00029	Liu et al., 2010	Yes
MGG_05059	Scytalone dehydratase MoRsy1	0.64	0.004197	Romao and Hamer, 1992	No
MGG_04489	Non-histone chromosomal protein 6 MoMnh6	0.74	0.001002	Lu et al., 2007	No
MGG_10447	Peptidyl-prolyl cis-trans isomerase MoCyp1	0.75	0.011926	Viaud et al., 2002	Yes
MGG_02252	Tetrahydroxynaphthalene reductase MoBuf1	0.83	3.24E-05	Romao and Hamer, 1992	Yes
MGG_09560	Exportin-5 MoExp5	0.83	0.033913	Tucker et al., 2010	Yes
MGG_02531	Minor extracellular protease MoVpr	1.25	0.027293	Guo et al., 2011	Yes
MGG_06148	Multifunctional β-oxidation protein MoMfp1	1.26	0.006355	Wang et al., 2007	Yes
MGG_01230	Succinate-semialdehyde dehydrogenase MoSsadh	1.29	0.016475	Guo et al., 2011	Yes
MGG_00063	Amyloglucosidase MoAgl1	1.33	0.000408	Badaruddin et al., 2013	Yes
MGG_06011	Glutathione Dehydrogenase MoSfa1	1.33	0.012853	Zhang et al., 2015b	Yes
MGG_01662	4-aminobutyrate aminotransferase MoAat	1.36	0.001644	Guo et al., 2011	Yes
MGG_04210	Arginine biosynthesis MoArg7	1.56	0.007287	Zhang et al., 2015a	Yes

^(a)^ Yes indicates that at least one potential N-glycosylation site was predicted in the amino acid sequence of protein while No stands for no potential N-glycosylation site was predicted in the amino acid sequence of protein.

**Figure 6:**
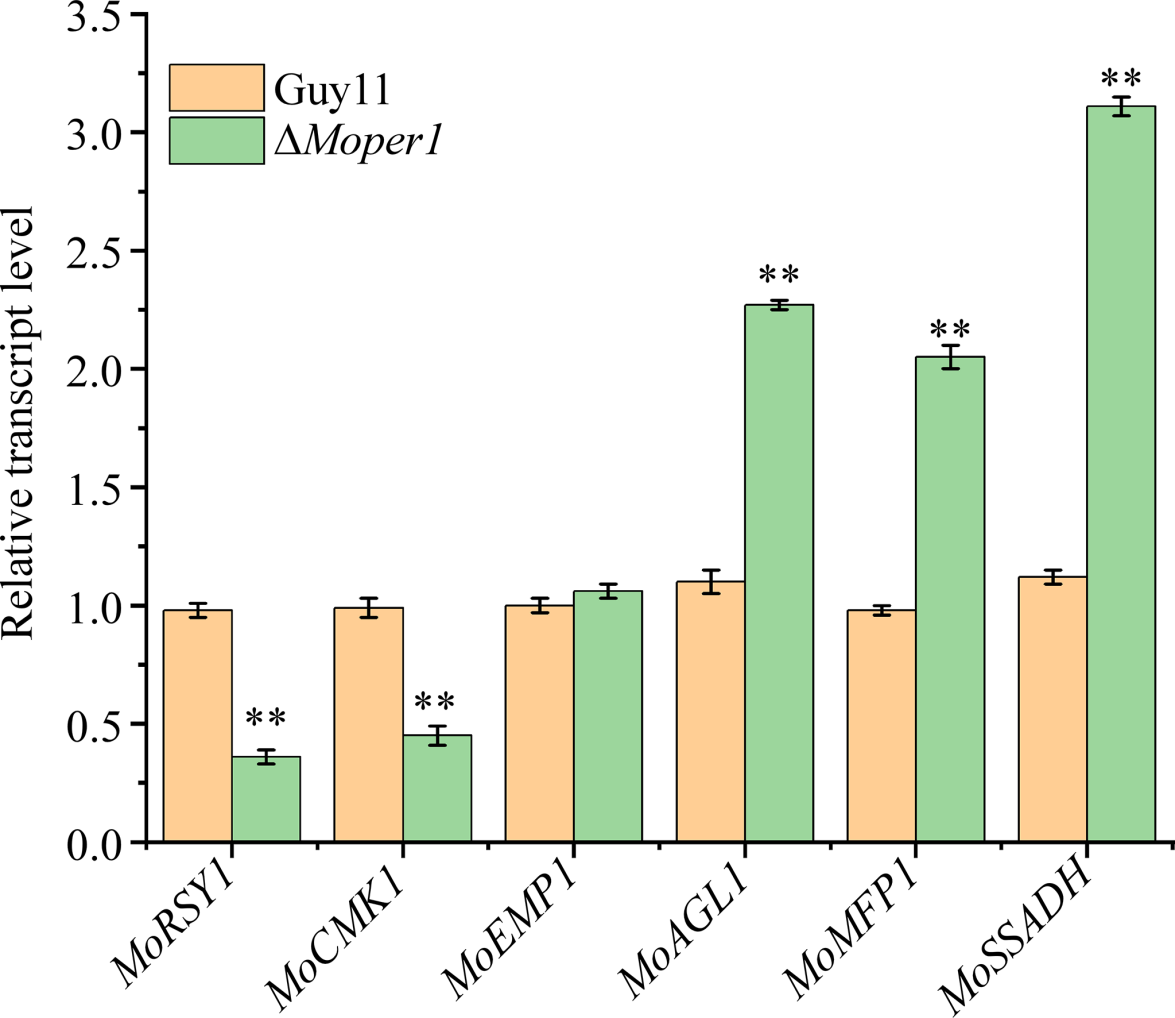
The expression fold changes at the mRNA levels of six DEPs that were involved in pathogenicity. Double asterisks respresent significant differences (*p* < 0.01).

To verify the reliability of proteomic data, we selected 3 downregulated and 3 upregulated genes encoding the above pathogenic essential proteins to verify the results of the proteome, and the results indicated that *MoRSY1* and *MoCMK1* genes were significantly downregulated, and that *MoAGL1*, *MoMFP1*, and *MoSSADH* genes were significantly upregulated in the ΔMoper1 mutant, which showed good correlation with protein abundance (**Figure 6**). The expression of MoEmp1 protein decreased by 52% in the proteomic data however, the expression of its encoding gene was not different between the Δ*Moper1* mutant and wild type (**Figure 6**).

## Discussion

As reported, after the *MoPER1* gene was knocked out or its expression was inhibited, the fungi was defective in growth, pathogenicity, and cell wall integrity, which indicated that MoPer1 plays an important role in the growth, development and pathogenicity of *M. oryzae* (Chen et al., 2018; Fan et al., 2019). To investigate MoPer1 protein’s effect mechanism in *M. oryzae*, we applied the TMT quantitative and qualitative analysis technique to examine the DEPs between the Δ*Moper1* mutant and Guy11.

According to our GO and KEGG analysis, these DEPs were involved in important biological functions. In the GO classification, DEPs were distributed in all three items and mostly associated with cell process, biological regulation, membrane, metabolic process, and molecular binding activity, and they are associated with growth and development, as well as pathogenicity of *M. oryzae* in a direct or indirect manner. In the KEGG pathway, we found that DEPs were involved in genetic information processing and metabolism, which was similar to the function in the GO classification. In particular, there were 8 DEPs that belonged to glycan biosynthesis and carbohydrate metabolism, indicating that deletion of *MoPER1* effected the expression of the related genes. Considering that MoPer1 is a GPI phospholipase and involved in GPI-anchored protein modification, we speculate that MoPer1 is involved in both carbohydrate metabolism and glycoprotein modification, thereby participating in cell wall integrity.

The present work identified 431 DEPs, greatly affecting ribosomal protein expression, which have a critical effect on protein translation and ribosomal assembly. Notably, great attention has been paid to such ribosome-independent activities. Furthermore, the above ribosomal proteins also relate to different pathophysiological processes (Zhou et al., 2015) this also has a great influence on the biological process of the rice blast fungus. Sorted by list hit data, knocking out *MoPER1* also has a huge impact on *in vivo* translation, and protein synthesis regulation plays a key role in eukaryotic biology through modulating homeostasis, growth, stress responses, and eukaryotic initiation. The translation initiation factor (eIF1, eIF2, eIF2B, eIF3, and eIF4G) is generally suppressed in our database; eIF3 can organize the interactions between ribosome and initiation factor and is necessary in productive translation (Lee et al., 2015), eIF-2B and eIF-4G activities have been identified as the critical factors for initiating translation (Price and Proud, 1994; Das and Das, 2016). Mitochondrial electron transport chain: Oxidative phosphorylation and oxidant production play an important role in the biological process. In the mitochondrial electron transport chain, diverse electron transfer reactions are employed for generating cell ATP by oxidative phosphorylation, MoPer1 is strongly correlated with energy metabolism, the production of ATP and NADH dehydrogenation program in the Δ*Moper1* mutant is affected, and upregulation of *NDUFS3* affects cell metabolism. As *MoPER1* gene is knocked out, it also affects other pathways like amino acid metabolism, lipid metabolism, and carbohydrate metabolism.

Among these regulated pathogenic essential proteins, their corresponding coding genes have critical effects on *M. oryzae* growth, development, and pathogenicity. In previous studies, Δ*Momnh6* and Δ*Movpr* decreased colony diameter, indicating that the deletion of these two genes affected the growth of *M. oryzae* (Lu et al., 2007; Guo et al., 2011). MoSfa1, MoCmk1, MoMnh6, MoArg7, MoRsy1, MoBuf1, and MoSsadh were required for conidiation, especially knockout of *MoSSADH*, which completely blocked conidial production (Romao and Hamer, 1992; Lu et al., 2007; Liu et al., 2010; Guo et al., 2011; Zhang et al., 2015a; Zhang et al., 2015b). More importantly, these 14 proteins were all involved in the pathogenicity on rice, and the reasons for the decreased pathogenicity of these mutants were mainly related to the reduction of appressorium formation ratio, the reduction of turgor pressure, the influence of appressorium melanin formation, etc. Except for *MoEMP1*, the transcript level of genes was more consistent with the proteome. As is well known, transcript level is usually, but not always, related to protein abundance. Consequently, qPCR results for those six genes conformed to TMT analysis indirectly. Considering that MoPer1 is a GPI phospholipase and potential N-glycosylation sites were predicted in 12 regulated proteins, we speculate that MoPer1 may function on some glycosylation-modified proteins to regulate the expression of these pathogenic essential proteins, and ultimately affected the normal growth, conidiogenesis, and pathogenicity of the fungi.

To summarize, we performed a quantitative proteomic analysis of TMT markers to identify the proteins regulated by MoPer1, and our results help us to further understand the molecular mechanism of MoPer1 regulating *M. oryzae* growth, development, and pathogenicity.

## Data Availability Statement

The datasets presented in this study can be found in online repositories. The names of the repository/repositories and accession number(s) can be found in the article/**Supplementary Material**.

## Author Contributions

YC, DZ, and YL conceived the project. YC, XW, CC, QH, CL, XZ, and XT prepared the samples and performed the research and analyzed the data. YC and XW were major contributors in writing the manuscript. DZ and YL revised the manuscript. All authors contributed to the article and approved the submitted version.

## Funding

This research was supported by the Natural Science Foundation of Hunan Province (2020JJ3024), the Science and Technology Innovation Program of Hunan Province (2021RC3114), the Natural Science Foundation of China (31972323), and the Agricultural Science and Technology Innovation Program of Hunan Province (2021CX35 and 2021CX71).

## Conflict of Interest

The authors declare that the research was conducted in the absence of any commercial or financial relationships that could be construed as a potential conflict of interest.

## Publisher’s Note

All claims expressed in this article are solely those of the authors and do not necessarily represent those of their affiliated organizations, or those of the publisher, the editors and the reviewers. Any product that may be evaluated in this article, or claim that may be made by its manufacturer, is not guaranteed or endorsed by the publisher.
