# Supplementary material for: Proteomics Analysis Reveals the Molecular Mechanism of MoPer1 Regulating the Development and Pathogenicity of Magnaporthe oryzae
Source: Front Cell Infect Microbiol. 2022 Jun 24;12:926771. doi: 10.3389/fcimb.2022.926771 (PMC9269092; doi:10.3389/fcimb.2022.926771)
Supplement: Supplementary file 3 [file Table_3.docx]

| Gene | Name | Primer for RT-qPCR |
| --- | --- | --- |
| Actin | FL4738 | CCATGTACCCTGGTCTTTCG |
|  | FL4739 | TTCGAGATCCACATCTGCTG |
| MGG_05059  *MoRSY1* | FL4740 | CAGAGAGCTCGTTCTAGGTTAC |
|  | FL4741 | TCTTTGAGAAGCTCAGTGGTTG |
| MGG_09912  *MoCMK1* | FL4742 | TTATTCTCCGTTCCGATCTGAG |
|  | FL4743 | CCTGATAAAGTTCTTTGCGTCC |
| MGG_00527  *MoEMP1* | FL4744 | TGAAGTTCACTCTTTCTGCTTG |
|  | FL4745 | GAAGTTGCTGTTGGTGAATTGG |
| MGG_00063  *MoAGL1* | FL4746 | CTGGCGCTTATGCCTTCTAC |
|  | FL4747 | CCCAGTCATTGGGGTATTTG |
| MGG_06148  *MoMFP1* | FL4748 | TATCATCAACAACGCCGGTA |
|  | FL4749 | AGCTCGTGAGAAACCAAGGA |
| MGG_01230  *MoSSADH* | FL4750 | TCGACCCTGAAACTACCCAC |
|  | FL4751 | AAGTTTCCTCTCGCGCAATC |

Suppl Table 3. Primer for qPCR validation
